# Effect of gender on the association between weight status and health-related quality of life in adolescents

**DOI:** 10.1186/1471-2458-12-997

**Published:** 2012-11-18

**Authors:** Emilie Bonsergent, Joseph Benie-Bi, Cédric Baumann, Nelly Agrinier, Sabrina Tessier, Nathalie Thilly, Serge Briançon

**Affiliations:** 1 University of Lorraine, Paris Descartes University, EA4360, Apemac, Nancy, France; 2 University of Lorraine, Faculty of Medicine, School of Public Health, Nancy, France; 3 Nancy University Hospital, Department of Clinical Epidemiology and Evaluation, Nancy, France; 4 UMR U557 INSERM/U1125 Inra/CNAM/Paris 13, SBMH-Paris 13 University, Bobigny, France

**Keywords:** Health-related quality of life, Weight status, Adolescents, High school

## Abstract

**Background:**

Some studies have investigated the association between body mass index (BMI) and health-related quality of life (HRQoL) among adolescents, but their results have been discrepant and few paid attention to the role of gender. The present investigation aimed to assess the relationship between weight status and HRQoL in adolescents and to verify whether it was similar in boys and girls.

**Methods:**

Five thousand two hundred and twenty six adolescents aged 14 to 18 years were included in the PRomotion de l’ALIMentation et de l’Activité Physique (PRALIMAP) trial, a 2x2x2 factorial cluster randomized trial performed in 24 high schools in France. Sociodemographic, anthropometric and HRQoL data were collected. BMI was categorized in four classes (thin, normal-weight, overweight, obese). Linear regression models were used to estimate the association between weight status and HRQoL, adjusting for confounders.

**Results:**

The mean age of adolescents was 15.7±0.6 years and their mean BMI was 21.6 ±3.5 kg/m^2^; 55% were girls. Boys were more often overweight and obese than were girls (overweight: 15.6% vs 14.2%, obese: 4.8% vs 3.3%), and girls were more likely to be thin (5.5% vs 4.5%, p=0.0042). All HRQoL scores were higher for boys (p=<0.0001). Weight status was not associated with physical and social scores neither in boys nor in girls. Conversely, it was associated with mental score, but differently in girls than boys. As compared with normal-weight girls, thin girls had better mental HRQoL (***β***=+6.17, p=0.0010), and overweight and obese girls had lower mental HRQoL (***β***=−3.89 and β=−5.90, respectively, p<0.001). Mental HRQoL was lower for thin, overweight and obese boys than for normal-weight boys (β= −4.97, β= −1.68 and β= −3.17, respectively, p<0.0001).

**Conclusions:**

Gender can modify the association between weight status and HRQoL in adolescents. Body image could be an important target of public health programs to improve subjective health during adolescence.

## Background

Obesity is a worldwide problem affecting an increasing number of people in all age groups, particularly children and adolescents [1], with differences in prevalence between boys and girls [2,3]. In France, the prevalence of overweight and obesity, classified according to the International Obesity Task Force [4], increased from 10.8% (overweight: 8.4%, obesity: 2.4%) to 15.7% (overweight: 12.4%, obesity: 3.3%) between 1990 and 2004 in adolescents aged 14 to 15 years [5]. A national study of nutrition and health reported a prevalence of overweight and obesity of 16.7% (overweight: 12.4%, obesity: 4.3%) in 2006 among adolescents aged 15 to 17 years [6].

Overweight and obesity can affect adolescent health, and metabolic and physiological changes associated with overweight and obesity in adolescence continue into adulthood and increase the risk of morbidity (e.g., coronary heart disease, diabetes mellitus and atherosclerosis) and mortality [7-11]. However, the consequences are not limited to physical health, as psychological and social aspects of well-being may also be affected [12].

Health-related quality of life (HRQoL) is a multidimensional concept that can be defined as an individual’s satisfaction or happiness in various life domains that affect or are affected by health [13-15]. During recent decades, HRQoL has become an important outcome to measure when assessing the effects of numerous disorders, short- and long-term disabilities, and diseases in different populations, and can help guide policies or interventions to improve health. Moreover, being overweight or obese in adolescence can affect future HRQoL [16]. Thus, improving HRQoL has become the primary justification for many interventions, medications, behavioural counselling programs, and other therapies [16,17]. Some studies suggest that there are differences in HRQoL between boys and girls during adolescence, with girls scoring lower than boys [18-20]. Previous studies in adolescents have also shown that overweight and obesity impair HRQoL [21-26], but the results have been discrepant. Some studies reported the greatest impairment in the physical and social dimensions of HRQoL [25], whereas others reported impairment in the emotional or mental dimensions [23,26].

The discrepant results may be explained by heterogeneous target populations (more often clinical- than community-based), and differences in study design and outcome measures (use of country-specific centile charts to define weight status and different HRQoL instruments) [25]. Although important associations were frequently found in clinical samples, such samples are often subject to selection bias (e.g. clinical samples more often present serious health conditions and lower HRQoL scores than do general population samples). Few studies have targeted the general population despite the potential interest in terms of the external validity of the results [25]. In addition, few studies investigated and showed differences between boys and girls in community samples; these studies also excluded thin adolescents [25] and only one looked at the possible modifying effect of gender on the association between body mass index (BMI) and HRQoL [26]. In fact, because thinness represents the ideal body image, mainly for females, the impact of weight status on HRQoL could differ between genders [27].

The present investigation aimed to assess the association between weight status and HRQoL in adolescents and to verify whether it was similar in boys and girls. The hypothesis that weight status influences HRQoL differently in boys and girls was tested.

## Methods

### Design

The present investigation was performed using a sample of 5,226 high school adolescents aged 14 to 18 years recruited for the PRALIMAP (PRomotion de l’ALIMentation et de l’Activité Physique) trial [28]. Body size and HRQoL data collected at inclusion in this trial were used. PRALIMAP was a 2x2x2 factorial cluster randomized trial assessing the effectiveness of three interventional strategies for overweight prevention (education, the environment, and overweight screening and care management) in 24 state-run high schools in France over 2 years. Data were collected at three visits: start of grades 10 (T0), 11 (T1) and 12 (T2). Adolescents were given written and oral information in the high school, and an information letter was sent to their parents. Adolescents whose parent(s) refused in writing did not contribute to data collection.

The trial was approved by the French ethics committee "Commission Nationale de l’Informatique et des Libertés" (n°906312), and registered in clinicaltrials.gov (no. NCT00814554; http://clinicaltrials.gov/ct2/show/NCT00814554).

### Data collection

Socio demographic, anthropometric and HRQoL data were collected at PRALIMAP inclusion in grade 10 (T0). Data on socio demographic characteristics and HRQoL were collected by self-administered questionnaires completed in the classroom. Additional socio demographic characteristics were obtained from the Board of Education database. Anthropometric measurements were performed by trained high school nurses.

#### Socio demographic data

Data were collected on age, gender, type of high school (general and technological or vocational), social and professional class of the family head (according to the definition of the national institute of statistical and economic studies in France [29]), parent occupation (how many parents work), adolescent perceptions of parental weight status (overweight or not) and family income (high, moderate or low). Age was classified in three groups according to the average age of adolescents registered for grade 10 in France (15 years): < 15 years, 15 years, > 15 years.

#### Weight status

Body weight and height were measured twice in a single session. Body weight of adolescents wearing underwear was measured to an accuracy of 0.05 kg using a calibrated electronic scale (SECA®: model number 873 1321009). Height was measured to the nearest 0.1 cm, without shoes, using a stadiometer (SECA®: reference SECA 214 SEC 01). BMI was calculated as weight/height^2^ from the means of the two measurements. The International Obesity Task Force (IOTF) and the Cole age- and sex-specific cut-off values for BMI were used to define four BMI classes: thin [30], normal, overweight and obese [4].

#### HRQoL

HRQoL was assessed using the Duke Health Profile for adolescents [31,32], a 17-item, generic, self-reporting questionnaire validated in the French language [32,33]. Among the 10 dimensions explored in this questionnaire, the physical, mental and social dimensions (five items each) are of most interest because they correspond to the World Health Organization (WHO) definition of health [34]; their scores are estimated independently on a 0–100 scale, with higher scores indicating better HRQoL.

### Statistical analysis

Continuous variables are described as means ± standard deviation (SD), and categorical variables as percentages. Because of the cluster design of the trial (cluster=high school), the intra-cluster similarity of HRQoL scores was estimated using the intraclass correlation coefficient (ICC) [28]. As the ICC was low (between 0.005 and 0.052), non-hierarchical analysis was used [35]. The comparisons between boys and girls were made using the Student *t* test for continuous variables, and the chi-square test for categorical variables. The interaction between gender and weight status was tested for all HRQoL dimensions. A deviation from linearity between BMI as a continuous and ordinal variable and HRQoL scores was observed (p<0.05) Thus BMI was considered as a nominal variable. Bivariate and multivariate analyses of variance models were conducted to estimate the association between weight status and HRQoL scores with HRQoL as the continuous dependent variable and weight status as a categorical main independent variable.

All statistical analyses involved use of SAS 9.2 (SAS Inst., Cary, NC, USA); p<0.05 was considered statistically significant.

## Results

### Sample characteristics

Characteristics of the 5,226 adolescents are shown in Table 1. The mean age was 15.7±0.6 years, and 55% were girls (n= 2,872). Both parents of 64.9% (n= 3,355) were working, and in 48.0% (n= 2,467) the family head was an employee or worker. Most adolescents attended general and technological high school (83.2%, n= 4,450). More than half reported a high family income level (n= 3,017) and 40.2% considered their parents overweight (n= 2,072).

**Table 1:** Sociodemographic characteristics weight status and health related quality of life (HRQoL) scores in adolescents in PRALIMAP

	**All N=5,226**		**Boys N= 2,354(45.0%)**		**Girls N= 2,354(45.0%)**		**p-value**
	**Mean/%**	**SD**	**Mean/%**	**SD**	**Mean/%**	**SD**	
**Socio demographics Characteristics**							
Age (years)	15.7	0.6	15.7	07	15.6	0.6	0.0004
Age groups							<0.0001
15 years old	68.0		64.8		70.5		
<15 years old	2.4		2.4		2.5		
>15 years old	29.6		32.7		27.0		
Type of high school							<0.0001
General/technological	83.2		78.2		87.2		
Vocational	16.8		21.8		12.8		
Social and professionals class of family head							0.0995
Farmers, store keeper, craftsmen, managers	8.2		7.7		8.6		
Executives	14.3		15.2		13.5		
Intermediate jobs	20.2		20.5		19.9		
Employees jobs	48.0		48.1		47.9		
Unemployed, retired	9.4		8.5		10.2		
Parent occupation							0.0100
Both parents work	64.9		66.1		63.9		
One parent has a job	29.6		29.5		29.8		
Neither parent work	5.4		4.4		6.3		
Perception of family income							0.7371
Low	6.4		6.2		6.6		
Moderate	34.2		34.7		33.9		
High	59.3		59.1		59.5		
Parents considered overweight by adolescent	40.2		37.9		42.1		0.0023
**Weight status**							
Body mass index (kg/m^2^)	21.6	3.5	21.5	3.5	21.6	3.5	0.1639
Body mass index class							0.0042
Thin	5.0		4.5		5.5		
Normal	76.1		75.1		77.0		
Overweight	14.9		15.6		14.2		
Obese	4.0		4.8		3.3		
**HRQoL scores (0-100)**							
Mental score	64.5	23.3	73.3	20.0	57.3	23.3	<0.0001
Physical score	75.6	18.5	82.2	15.7	70.1	18.8	<0.0001
Social score	68.8	19.1	71.9	18.7	66.2	19.1	<0.0001

a: Chi-square test for qualitative variables; Student test for qualitative variables; b: according to the international Obesity Task Force.

As compared with girls, boys were older (p=0.0004), more often attended a vocational high school (p<0.0001), less often considered their parents as overweight (p=0.0023) and were more likely to have two parents working (p=0.0100) (Table 1).

### Weight status and HRQoL

The mean BMI was 21.6±3.5 kg/m^2^ and did not differ significantly between genders (Table 1). The prevalence of overweight and obesity was higher for boys than girls (overweight: 15.6% vs 14.2%, obese: 4.8% vs 3.3%), whereas the proportion of those being thin was higher among girls than boys (5.5% vs 4.5%) (p=0.0042). Physical HRQoL scores were higher than social and mental scores. Boys had significantly higher HRQoL scores than girls, whatever the dimension (all p<0.0001, Table 1).

### Effect of gender on the association between weight status and HRQoL

For both boys and girls, the higher the age, the lower the HRQoL scores. Moreover, the less favourable the socio demographic parameters (social and professional class, parental occupation, perception of family income and of parental weight status), the lower the HRQoL scores (Tables 2 and 3).

**Table 2:** Bivariate and multivariate analysis of the association between weight status and HRQoL in boys (N=2,354)

**Variables**	**Mental HRQoL**				**Physical HRQoL**				**Social HRQoL**			
	**Bivariate**		**Multivariate**		**Bivariate**		**Multivariate**		**Bivariete**		**Multivariate**	
	**β**	**SE**	**β**	**SE**	**β**	**SE**	**β**	**SE**	**β**	**SE**	**β**	**SE**
**Weight status**^**#**^**(vs normal)**												
Thin	−4.72	2.01	−4.91*	2.02	−1.85	1.57	−2.33^NS^	1.60	−3.96	1.88	−3.79 ^NS^	1.89
Overweight	−2.51	1.14	−1.79	1.16	−0.92	0.90	−0.49	0.91	−1.17	1.07	−0.81	1.08
Obese	−5.04	1.93	−3.63	1.97	−1.84	1.51	−0.46	1.56	−3.10	1.80	−2.80	1.84
**Sociodemographic characteristic**												
Vocational high school (vs general and technological school)	-1.28	1.02	−0.16^NS^	1.12	−1.47	0.80	−0.24 ^NS^	0.88	−0.44	0.95	0.36 ^NS^	1.05
Age groups (vs 15 years old)												
<15 years old	−0.19	2.68	0.80***	2.68	1.18	2.10	1.37***	2.12	−1.36	2.52	−1.36 ^NS^	2.50
>15 years old	−5.04	0.88	−4.77	0.96	−3.68	0.69	−3.42	0.76	−2.37	0.82	−2.02	0.90
Social and professional class of family head (vs executives)												
Farmers, storekeepers, craftsmen, managers	1.85	1.85	2.43^NS^	1.85	0.24	1.44	1.03 ^NS^	1.46	0.90	0.71	1.62	1.73
Intermediate jobs	−1.21	1.41	−0.42	1.41	−1.40	1.10	−0.60	1.12	0.52	1.31	0.98	1.32
Employees, workers	−0.85	1.23	1.22	1.27	−1.85	0.96	−0.25	1.00	0.84	1.14	2.37	1.18
Unemployed retired	−2.66	1.79	0.71	2.02	−3.19	1.40	−0.69	1.59	−4.49	1.66	−1.96	1.88
Parent occupation (vs both parents work)												
One parent has a job	−1.62	0.92	0.11^NS^	0.96	−1.31	0.72	−0.28 ^NS^	0.76	−2.23	0.86	−0.81 ^NS^	0.90
Neither parent works	−2.20	2.04	1.28	2.41	−1.53	1.60	1.23	1.90	−2.66	1.92	2.59	2.26
Perception of family income (vs perception of high income)												
Moderate	−3.64	0.89	−3.15***	0.91	−3.13	0.70	−2.76**	0.72	−4.01	0.82	−3.83	0.85
Low	−11.99	1.75	−11.12	1.82	−3.87	1.37	−3.31	1.44	−11.09	1.62	−10.5	1.70
Parents considered overweight (yes vs no)	−5.72	0.85	−4.97***	0.87	−3.10	0.67	−2.80***	0.69	−2.83	0.80	−2.19*	081

# International Obesity Task Force body mass index classes; *NS*: no significant; *p<0.05; **p<0.001; ***p<0.0001; *SE*: standard error.

**Table 3:** Bivariate and multivariate analysis of the association between weight status and HRQoL in girls (N=2,872)

**Variables**	**Mental HRQoL**				**Physical HRQoL**				**Social HRQoL**			
	**Bivariate**		**Multivariate**		**Bivariate**		**Multivariate**		**Bivariete**		**Multivariate**	
	**β**	**SE**	**β**	**SE**	**β**	**SE**	**β**	**SE**	**β**	**SE**	**β**	**SE**
**Weight status**^**#**^**(vs normal)**												
Thin	6.93	1.90	6.46***	1.91	2.47	1.55	2.16 ^NS^	1.57	−0.19	1.57	0.43 ^NS^	1.57
Overweight	−4.86	1.24	−3.45	1.24	−0.85	1.01	0.41	1.02	−2.58	1.03	−1.94	1.02
Obese	−8.49	2.43	−5.24	2.47	−3.33	1.98	−0.78	2.02	−2.70	2.02	0.88	2.04
**Sociodemographic characteristic**												
Vocational high school (vs general and technological school)	−4.88	1.43	−1.85 ^NS^	1.55	−4.30	1.16	−3,49 ^NS^	0,72	−5.76	1.18	−3.28*	1.27
Age groups (vs 15 years old)												
<15 years old	1.53	2.78	2.17	2.74	0.58	2.25	0.94***	2.24	−2.65	2.27	−3.04***	2.25
>15 years old	−7.10	0.97	−6.35	1.03	−4.48	0.79	−3.91	0.85	−5.34	0.80	−3.76	0.85
Social and professional class of family head (vs executives)												
Farmers, storekeepers, craftsmen, managers	−1.70	1.91	0.44 ^NS^	1.87	0.44	1.54	1.52 ^NS^	1.53	−1.81	1.56	0.08***	1.54
Intermediate jobs	−2.95	1.54	−0.36	1.52	−1.94	1.24	0.03	1.24	−1.44	1.26	0.31	1.25
Employees, workers	−3.97	1.35	0.35	1.36	−0.87	1.09	1.86	1.11	−2.49	1.10	0.80	1.12
Unemployed retired	−2.76	1.82	3.68	2.08	−3.17	1.46	1.34	1.71	−4.30	1.49	1.83	1.72
Parent occupation (vs both parents work)												
One parent has a job	−2.75	0.97	−0.89 ^NS^	0.99	−2.11	0.78	−0.79 ^NS^	0.82	−2.80	0.79	−1.12***	0.82
Neither parent works	−2.36	1.82	0.00	2.16	−1.68	1.48	1.37	1.77	−4.61	1.50	−1.11	1.78
Perception of family income (vs perception of high income)												
Moderate	−6.19	0.93	−5.75***	0.94	−4.95	0.75	−4.54***	0.77	−6.57	0.76	−6.20***	0.77
Low	16.57	1.76	−15.42	1.83	−10.57	1.44	−9.52	1.50	−13.61	1.44	−12.56	1.51
Parents considered overweight (yes vs no)	−5.33	0.88	−3.72***	0.88	−4.62	0.71	−3.49***	0.72	−3.69	0.72	−2.46**	0.73

#International obesity task force body mass index classes; *NS*: no significant*p<0,05; **p<0,001; ***p<0,0001.

*SE*: standard error.

**Figure 1:**
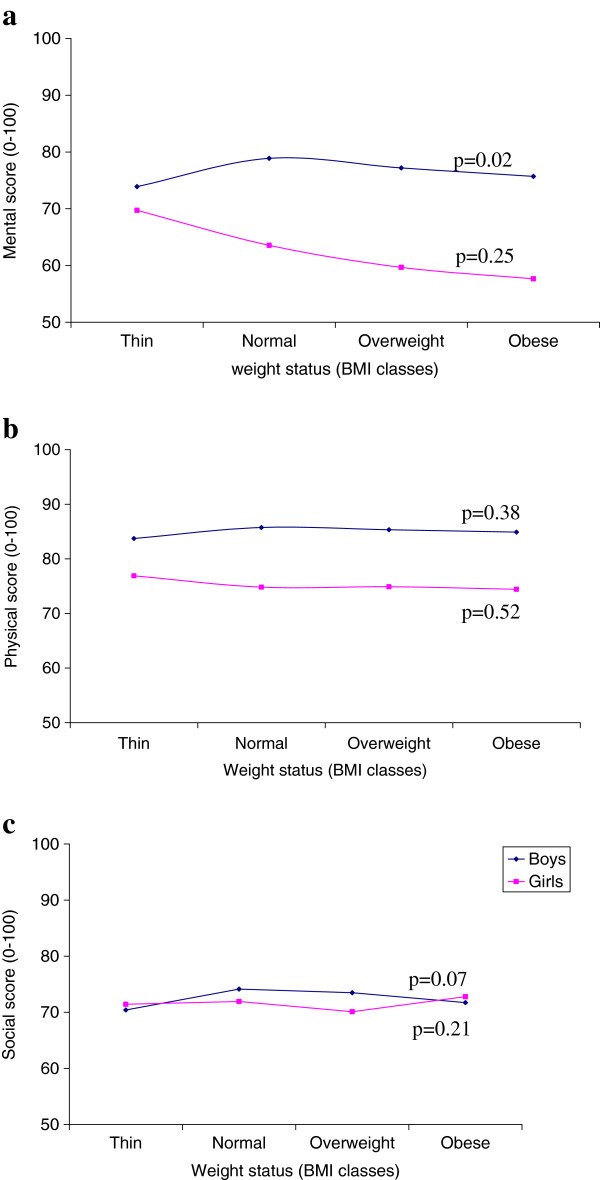
**Trends of mental (a), physical (b) and social (c) HRQoL scores according to weight status.** Note: Weight status is in BMI classes. Trends of HRQoL scores are adjusted for confounders. BMI classes are defined according to the International Obesity TaskForce (IOTF) classification; p: p-value of the linearity test of weight status on HRQoL scores (if p-value<0.05 then the linearity was not verified).

On multivariate analysis, weight status was associated with mental HRQoL but not with physical and social HRQoL scores for boys and girls (Tables 2 and 3). A significant interaction between gender and weight status (p<0.0001) was found; the higher the BMI, the lower the mental HRQoL score in girls, but not in boys (Figure 1). As compared with normal-weight girls, thin girls had a higher mental HRQoL score (β=+6.17, p=0.001), whereas overweight and obese girls had a lower mental HRQoL score (β=−3.89, p=0.016 and β=−5.90, p=0.0374 respectively). In contrast, thin, overweight and obese boys had a lower mental HRQoL score than normal-weight boys (β= −4.97, β= −1.68 and β= −3.17 respectively, p=0.0221).

## Discussion

Gender moderated the association between weight status and mental HRQoL score. Among girls, mental HRQoL was higher for thin girls and decreased with increasing BMI. In contrast, mental HRQoL was lower for thin, overweight and obese rather than normal-weight boys.

Only one study investigated the effect of gender on the association between weight status and HRQoL in a community sample including older adolescents (14–18 years old) [26] who are affected by physical and psychological changes due to puberty. Keating et al. showed that girls who were overweight and obese had significantly lower physical functioning scores than normal-weight girls, but the same was not true for boys. Similarly, girls who were obese had significantly lower school and emotional functioning scores than did normal-weight girls, but the same was not true in boys. Keating et al. excluded thin adolescents, included younger and older adolescents (11–18 years), and used the PedsQL to estimate HRQoL scores. In our study, thin adolescents were not excluded, only older adolescents were included (14–18 years), and the adolescent Duke Health Profile was used. Like Keating et al., the present investigation showed that overweight and obese girls had poorer mental HRQoL score than did normal-weight girls. However, weight status (including thin) and mental HRQoL score were linearly and negatively related in girls but in not boys, thin girls having a better mental HRQoL score than normal-weight girls. Thus, it appears important to include thin adolescents when investigating weight status.

Our results suggest that the perception of ideal body size and shape differs between adolescent girls and boys; thinness represented the ideal body image for girls, which could explain the better mental HRQoL score in thin girls [27]. The representation of the ideal body shape may come from a collective vision influenced by television, magazines, advertisements and the social stigma attached to obesity and the “fat phobia” that pervades our daily life. Girls seek to be thin and fit, not only to be healthy but also to be perceived by themselves and others as having desirable personal qualities. Girls may be more attuned to or aware of their bodies and their health than boys. Girls consistently report greater body dissatisfaction than do boys [36,37]. Some studies showed that girls were more likely to perceive themselves as being overweight than boys [36,38-40]. In a study by Lawler et al. [41], all overweight girls revealed a desire to weigh less, as compared with only 78.6% of overweight boys. Average-weight girls wanted to be lighter, whereas average-weight boys were satisfied with their bodies or wanted to be bigger [41]. Boys find a greater variety of body shapes socially acceptable compared to girls, and girls have a narrower range of what is considered the ideal body image [42]. Even if some men strive to lose weight to conform to today’s ideal body shape, the ideal image for most boys is muscular and strong, the main characteristics of virility, so boys with an athletic build may be more popular among their peers and more difficult to victimize [43]. This observation may explain the non-linear association between BMI and HRQoL observed in boys.

Cross-sectional community surveys reflect the subjects’ HRQoL at one time, which means that temporality cannot be established [44] and it remains unclear whether BMI determines HRQoL or vice versa. Some studies merged data for adolescents and younger children [25], but findings in children cannot be extrapolated to adolescents. Adolescence is the period of transition to adulthood and sexual differentiation. This stage presents several characteristics that justify a particular interest in terms of obesity, as well as at the level of prevention, screening and care. Indeed, obese adolescents are more likely than their normal-weight counterparts to remain obese into adulthood (78% for men vs. 63% for women) [45]. Moreover, the large sample size gave the study considerable statistical power. In addition, the quality of anthropometric measurements reported by qualified and trained nurses in our study attested to the validity of the data and minimized biased measurements. As linearity was not verified, BMI was considered as o nominal variable with four classes including thinness. Therefore, HRQoL score was estimated for thinness, and strongly differed between girls and boys.

### Implications for research and public health

The association between weight status and HRQoL in adolescents must be studied by gender and the results taken into account when developing health programs, in order to identify the best strategy with which to address these issues. The present investigation found that girls had significantly lower mental HRQoL than did boys and their decrease in HRQoL was higher when their body size increased. This information could help educators implementing health programs that focus on the educational system and are tailor-made to meet the specific needs of target groups. Finally, thinness in boys affected HRQoL in the same way as overweight and obesity in boys. Thinness may be an issue to consider in public health programs for adolescents.

## Conclusions

Gender moderates the association between weight status and HRQoL mental dimension among adolescents in the PRALIMAP trial, with a linear decrease in girls and a non-linear decrease in boys. Body image could be an important target of interventions to improve subjective health in adolescence. The present study contributes to the knowledge of adolescent HRQoL by providing a better understanding of gender-specific factors associated with body image and highlighting the principal role of weight status in HRQoL for both boys and girls. Targeting educational interventions to adolescents could be helpful in reducing the increasing prevalence of obesity and the effect of overweight on mental HRQoL. Public health programs must take this into account in order to adapt their message to adolescents. Longitudinal studies would be helpful in order to establish the causality of the association between BMI and HRQoL in adolescents.

## Abbreviations

BMI: Body Mass Index; HRQoL: Health-Related Quality of Life; ICC: Intra-class Correlation Coefficient; IOTF: International Obesity TaskForce; PedsQL: Pediatric Quality of Life inventory; PRALIMAP: PRomotion de l’ALIMentation et de l’ACtivité Physique; WHO: World Health Organization.

## Competing interests

The authors declare that they have no competing interests.

## Authors’ contributions

All the authors actively participated in the collaborative work leading to the publication. NA, EB, JB, CB, SB, ST and NT are outcomes evaluation and statistical managers. EB, JB, CB and SB drafted the manuscript. All the authors read and approved the final manuscript. EB is the paper guarantor.

## Pre-publication history

The pre-publication history for this paper can be accessed here:

http://www.biomedcentral.com/1471-2458/12/997/prepub
